# Task vs. rest—different network configurations between the coactivation and the resting-state brain networks

**DOI:** 10.3389/fnhum.2013.00493

**Published:** 2013-09-17

**Authors:** Xin Di, Suril Gohel, Eun H. Kim, Bharat B. Biswal

**Affiliations:** ^1^Department of Biomedical Engineering, New Jersey Institute of TechnologyNewark, NJ, USA

**Keywords:** brain network, coactivation, hub shift, meta-analysis, modularity, resting-state, small world, thalamus

## Abstract

There is a growing interest in studies of human brain networks using resting-state functional magnetic resonance imaging (fMRI). However, it is unclear whether and how brain networks measured during the resting-state exhibit comparable properties to brain networks during task performance. In the present study, we investigated meta-analytic coactivation patterns among brain regions based upon published neuroimaging studies, and compared the coactivation network configurations with those in the resting-state network. The strength of resting-state functional connectivity between two regions were strongly correlated with the coactivation strength. However, the coactivation network showed greater global efficiency, smaller mean clustering coefficient, and lower modularity compared with the resting-state network, which suggest a more efficient global information transmission and between system integrations during task performing. Hub shifts were also observed within the thalamus and the left inferior temporal cortex. The thalamus and the left inferior temporal cortex exhibited higher and lower degrees, respectively in the coactivation network compared with the resting-state network. These results shed light regarding the reconfiguration of the brain networks between task and resting-state conditions, and highlight the role of the thalamus in change of network configurations in task vs. rest.

## Introduction

The human brain exhibits organized spontaneous fluctuations in the resting-state (Biswal et al., 1995), enabling researchers to study large-scale brain segregations and integrations (Bullmore and Sporns, 2009, 2012; Menon and Uddin, 2010). The spontaneous fluctuations reveal high synchronization between brain regions in the same brain system (Cordes et al., 2000; Greicius et al., 2003), and are relatively independent between different brain systems (Beckmann et al., 2005; Biswal et al., 2010). The whole brain segregation and integration can also be studied using graph theory based analysis (Bullmore and Sporns, 2009; Wang et al., 2010). For example, the brain network in the resting-state revealed modular structures, small world and scale free properties (Salvador et al., 2005; Achard et al., 2006; Achard and Bullmore, 2007; Nakamura et al., 2009; Yan and He, 2011).

Despite the growing popularity of resting-state fMRI to study brain functions, studies have yet to address a fundamental question regarding whether the brain at resting-state is comparable to the brain during task performing. Given that the evoked cerebral blood flow by different tasks account for less than 5% of the resting-state cerebral blood flow (Raichle, 2010), the resting-state brain already represents a large proportion of hemodynamic information which may reflect brain maintenance. Studies have also shown that task-related coactivation patterns correspond well with the brain systems that are measured during the resting-state (Toro et al., 2008; Smith et al., 2009). However, based on the economic theory of brain network organization, the brain network should be in an energy saving mode during the resting-state, while exhibiting dynamic network reconfiguration in the presence of a task demand to facilitate global and between systems information transmissions (Bullmore and Sporns, 2012). We predict that even though the connectivity in task conditions and the resting-state may be similar, substantial differences of network configurations may take place to support different task demands.

Changes in connectivity modulated by task are important to understand brain integration (Friston, 2011). Specific connections have been shown to be modulated by specific tasks (McLntosh and Gonzalez-Lima, 1994; McIntosh et al., 1994; Büchel and Friston, 1997; Rao et al., 2008). However, the modulations of connectivity are task specific, and it is difficult to modulate the whole brain network using a specific task. Thus, we adopted the same approach as Toro et al., and Smith et al. to examine task activations or group differences and their corresponding coactivation pattern across the whole brain (Toro et al., 2008; Smith et al., 2009). Specifically, we constructed brain networks comprised of 140 regions of interest (ROIs) from the whole brain based on both meta-analytic coactivation patterns (Yarkoni et al., 2011) and resting-state correlations of fMRI signals (Biswal et al., 2010). The online database Neurosynth (http://old.neurosynth.org/) was used to extract coactivation information, which contained 47,493 activations from 4393 studies (Yarkoni et al., 2011). We first asked whether the strength of coactivation between a pair of ROIs was correlated with their resting-state correlations. We then compared different network properties based on graph theory between the two brain networks (Bullmore and Sporns, 2009), including the small-worldness (Watts and Strogatz, 1998), modularity (Newman, 2006), and hub distributions. We hypothesized that the brain when performing tasks will be more integrated and thus exhibit higher global efficiency and reduced modularity compared with the resting-state brain. In addition, we hypothesized that the brain hubs may shift from the default mode network (DMN) (Raichle et al., 2001; Greicius et al., 2003) regions to other brain regions that are critical during task executions.

## Methods

### Regions of interest

One hundred and sixty functionally defined ROIs from Dosenbach et al. were adopted in the present analysis (Dosenbach et al., 2010). Twenty-four ROIs were removed because they were outside the Neurosynth mask. We included four more ROIs that were not represented within the 136 ROIs (Sabatinelli et al., 2011): the right amygdala (Montreal Neurological Institute, MNI, coordinates: 20, −4, −15), the left amygdala (−20, −6, −15), the right parahippocampus (14, −33, −7), and the left parahippocampus (−20, −33, −4). A total of 140 ROIs were used in the present study to construct brain networks (supplementary Table S1).

### Coactivation network

The online database, Neurosynth, was used to construct the coactivation network (Yarkoni et al., 2011). The database search was conducted in November, 2012 when the database had 4393 studies and 47,493 activations. For each of the 140 ROIs, Neurosynth identified all the papers in the database that reported coordinates within 10 mm from the ROI center, and exported a whole brain z-score map representing the likelihood that a voxel coactivated with the given ROI (Yarkoni et al., 2011). The images were thresholded using a false discovery rate (FDR) criterion of *p* < 0.05. Thus, the Neurosynth search of all the ROIs resulted in 140 coactivation maps.

One hundred and forty spherical ROIs were defined using radii of 10 mm. The coactivation values of 140 ROIs were extracted from 140 coactivation maps, which resulted in a 140 × 140 matrix. Each row of the matrix represented the coactivation values of a given ROI with the other ROIs. Because the number of papers that was returned by each ROI inquiry was different, the coactivation values from different ROI inquiry may be biased. Therefore, we normalized each row by dividing the value from the ROI corresponding to that row, so that the diagonal values of the matrix were equaled to one. In addition, since the distribution of the coactivation values are skewed, all the values of the matrix were added by one, and were logarithmically transformed to facilitate a normal distribution. Finally, because the coactivation likelihood of region A with region B and the coactivation likelihood of region B with region A are generally similar but have slightly different values, the matrix was transposed, and averaged with the original matrix to create a symmetrical coactivation matrix.

### Resting-state network

We analyzed a resting-state fMRI data set to construct a resting-state network to compare with the coactivation network. The Oulu dataset from the 1000 Functional Connectomes Project was used (Biswal et al., 2010). This dataset originally contains 103 subjects. One subject's data was discarded because of large head motion (greater than 3 mm). Thus 102 subjects' data were included in the current analysis (36M/66F). The mean age was 21.5 years (range from 20 to 23 years). Two hundred and forty-five resting-state functional images were acquired for each subject (*TR* = 1.8 s, 28 slices). High resolution anatomical image was also acquired for each subject using MPRAGE sequence (Magnetization Prepared Rapid Acquisition Gradient Echo). More information for the data can be found at http://fcon_1000.projects.nitrc.org/fcpClassic/FcpTable.html. To rule out the possibility that the current results are due to sample bias of the resting-state dataset, we have conducted a separate analysis using another resting-state dataset, i.e., the Nathan Kline Institute (NKI) / Rockland Sample (http://fcon_1000.projects.nitrc.org/indi/pro/nki.html). Data analyses were identical to the Oulu dataset. Detailed methods and results of NKI dataset are reported in the supplementary material section.

Functional MRI images were processed using the SPM8 toolbox (http://www.fil.ion.ucl.ac.uk/spm/) under the MATLAB7.7 environment (http://www.mathworks.com). First, the MPRAGE anatomical image for each subject was segmented into gray matter (GM), white matter (WM), and cerebrospinal fluid (CSF) using the new segment routine in SPM8. The deformation field maps were also obtained in this step to later normalize the functional images. For each subject, the first five images of the fMRI images were discarded, resulting in 240 images per subject. The functional images were then motion corrected using the realign function. One subject's data were discarded after this step because the head motion was greater than 3 mm, resulting in 102 subjects in total. Next, the functional images were coregistered to the subjects' own anatomical images. Then, the deformation field map obtained from new segmentation step was applied to the functional images to normalize them into the standard MNI space.

One hundred and forty times series from the corresponding ROIs were extracted for each of the subjects. Six head motion parameters and their first order derivatives, first five eigenvectors from signals within WM masks, and first five eigenvectors from signals within CSF masks were regressed out using linear regression (n, ón, Ongür and Whitfield-Gabrieli). No global signal regression was applied. Next, the time series were temporally filtered using a band-pass filter of 0.01–0.1 Hz. For each subject, a 140 × 140 correlation matrix was calculated using Kendall's rank correlation to minimize spurs correlations due to noises. The correlation matrices were transformed into Fisher's z, and averaged across subjects. Finally, the mean Fishers' z matrix was transformed back to correlation matrix using Fisher's inverse transform.

### Network analysis

Because the values in the coactivation matrix and the resting-state correlation matrix are essentially different, network sparsity thresholds were used to keep the number of edges of the two networks the same when comparing the two networks. The sparsity range was set between 6 and 40% with an increment of 1%. This range was used because typical sparsity of human neuron network is between this range, and the large scale brain networks revealed small world properties within this range (Achard and Bullmore, 2007; He et al., 2008). After thresholding, all the networks were binary (unweighted) undirected networks.

We first compared the two networks in terms of small world properties (global efficiency and mean clustering coefficient) and modularity. The global efficiency characterizes how efficient the whole brain network integrates information, and the mean clustering coefficient characterizes how efficient the information flows around local nodes (Watts and Strogatz, 1998). Modularity, also known as Newman's Q, characterizes the extent the whole brain network can be divided into sub-communities (Newman, 2006). The global efficiency, mean clustering coefficient, and modularity were calculated for the two networks at each sparsity level using the brain connectivity toolbox (Rubinov and Sporns, 2010). As a reference, random networks were generated 1000 times at each sparsity level. The three parameters were also calculated for the random networks, and were averaged across the 1000 random networks.

To determine the statistical significance, we created a null distribution of network differences by randomly shuffling the two networks 1000 times and calculating their differences of network properties for 1000 random networks. Specifically, at each sparsity level, we first identified edges that were different between the two networks. Next, we randomly assigned 50% of these different edges from the coactivation network to the resting-state network, and vice versa, resulting in two new mixed networks. We then calculated the three network parameters, i.e., the global efficiency, mean clustering coefficient and modularity, for the two mixed networks and obtained their differences between the two networks. The randomizations were performed 1000 times for each sparsity level to obtain a difference distribution. The difference of the three parameters between the coactivation network and the resting-state network were then compared with the randomized distribution to determine statistical significances. A critical threshold of *p* < 0.001 was used.

To demonstrate modular structures of the coactivation and the resting-state network, we thresholded the two networks at a sparsity level of 20%, and entered the two unweighted undirected networks into Gephi (https://gephi.org/) to determine their modular structures using the algorithm by Blondel et al. (2008). The two networks and their modular structures were rendered into a 2D surface using the Fruchterman–Reingold Algorithm (Fruchterman and Reingold, 1991).

We then examined whether the two networks displayed similar hub distribution (Achard et al., 2006). In the present study, we simply defined the importance of each node by calculating the number of edges connected to this node (also known as degree). We calculated the degrees for each node for the two networks at each sparsity level. Next, at each sparsity level, correlations of node degrees between the coactivation network and the resting-state network were calculated at the sparsity range of 6–40%. The correlations reflected the similarity of hub distributions of the two networks. There were only small correlations of degrees between the two networks (see Results below), i.e., a high degree node in the resting-state network was not necessarily a high degree node in the coactivation network. Hence, we subtracted the degrees between the two networks for each node at the sparsity level of 10, 20, and 30%. At each of the three sparsity levels, we sorted the degree differences. The two networks were also randomized using the method mentioned above, and the sorted degree differences were calculated. The randomization was conducted 1000 times, and the distribution of the sorted degree differences was obtained. Then, the original sorted degree differences between the coactivation network and resting-state network was compared with the distribution. Next, at each sparsity level, five nodes that had the largest degree differences and five nodes that had the least degree differences between the coactivation network and the resting-state network were identified. These nodes were rendered on a brain surface model using the BrainNet Viewer (http://www.nitrc.org/projects/bnv/).

## Results

### Coactivation and resting-state networks

**Figure 1:**
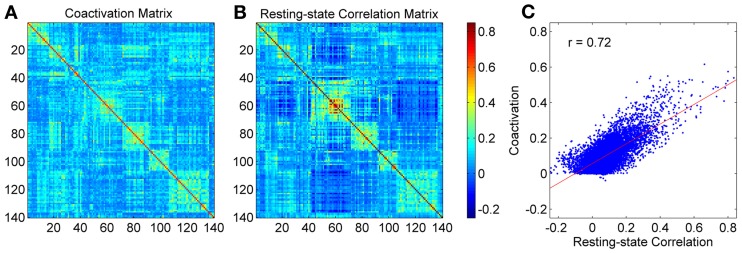
**Coactivation matrix (A), resting-state correlation matrix (B), and the relationship between coactivation strengths and resting-state correlations (C).** Each dot in the scatter plot represents one pair of ROIs. The red line in panel **(C)** represents the linear fit.

The pattern of the coactivation and the resting-state correlation matrix were comparable (Figures 1A,B). Because the ROIs were arranged according to their network affiliations as reported by Dosenbach et al. (2010), square like structures along the diagonal were observed in both networks (see supplementary Table S1 for the network affiliations of the ROIs). In addition, the coactivation strengths and the resting-state correlation strengths among the 9730 (140 × 139/2) pairs of ROIs showed a strong linear relationship (Figure 1C), i.e., if two regions had higher correlation in the resting-state, they also had higher coactivation strength, and vice versa. The Pearson correlation between the coactivation strengths and connectivity strengths was 0.72 (*r*^2^ = 0.51).

### Small world and modularity

**Figure 2:**
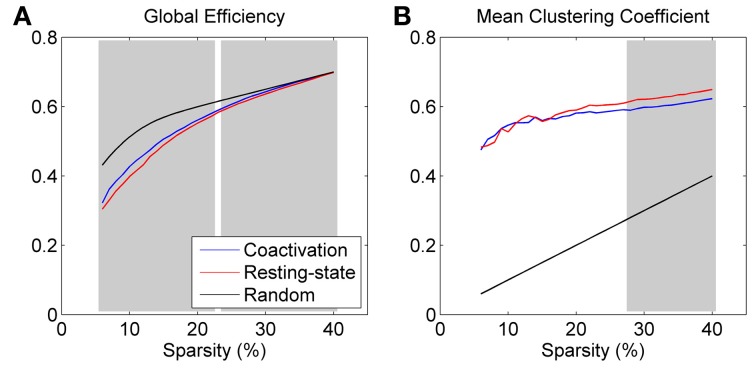
**Global efficiency (A) and mean clustering coefficient (B) for the coactivation, resting-state, and random networks as a function of connectivity sparsity.** The shading areas represent significant differences between the coactivation and resting-state networks at *p* < 0.001 based on 1000 permutations.

Both the coactivation network and the resting-state network revealed smaller global efficiency and larger clustering coefficient compared with the reference random networks, which characterizes the small world network properties (Figure 2). Direct comparison between the coactivation network and the resting-state network revealed greater global efficiency and smaller mean clustering coefficient for the coactivation network compared with the resting-state network at selected sparsity levels (highlighted by shading in Figure 2). Thresholding at a significance level of *p* < 0.001, greater global efficiency for the coactivation network were present at almost all the sparsity levels that were tested between 6 and 40% (except for 23%), while smaller mean clustering coefficient for the coactivation network were only present at the sparsity level between 28 and 40%.

**Figure 3:**
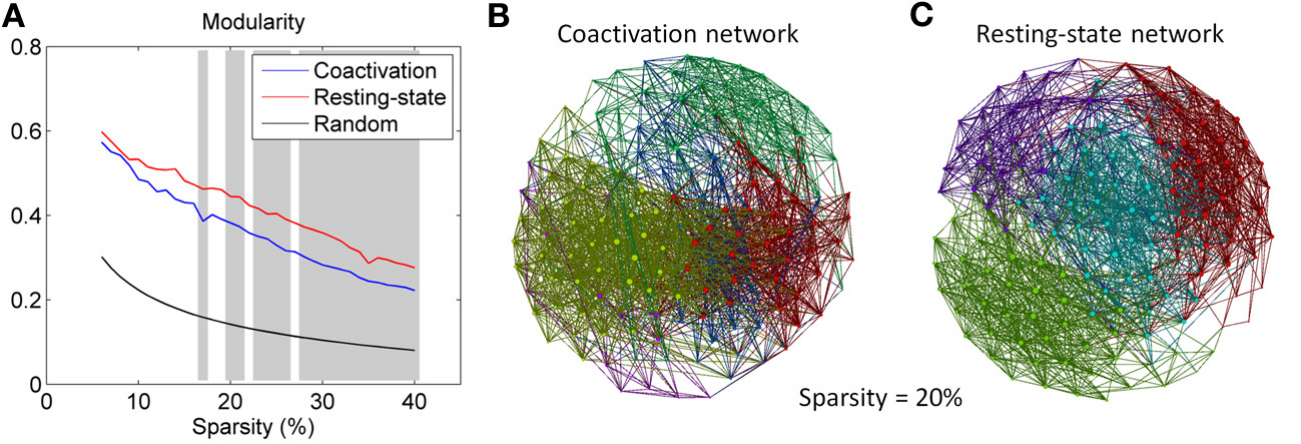
**(A)** Modularity for the coactivation, resting-state, and random networks as a function of connectivity sparsity. The shading areas represent significant differences between the coactivation and resting-state networks at *p* < 0.001 based on 1000 permutations. Panels **(B,C)** demonstrate the modular structures for the coactivation network and the resting-state network at sparsity level 20%. The node colors in panels B and C encode different modules.

Both the coactivation network and the resting-state network revealed higher modularity compared with the random networks (Figure 3A). The coactivation network generally revealed lower modularity than the resting-state network at sparsity level between 17 and 40% at the significance level of *p* < 0.001. Figures 3B,C demonstrated the modular structures of the coactivation network and the resting-state network at sparsity level of 20%. For the resting-state network, four modules were clearly visible with a large number of within module connections and a small number of between modules connections. In contrast, five modules for the coactivation network were difficult to distinguish since there were large numbers of between module connections.

### Hub shifts

**Figure 4:**
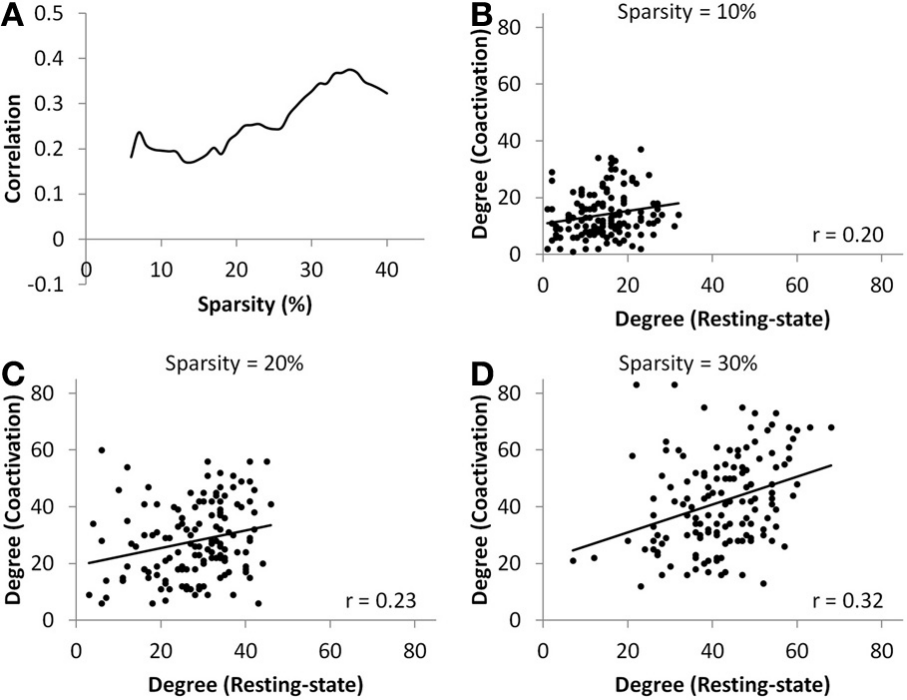
**(A)** Correlations between nodes' degree of the coactivation network and the resting-state network as a function of connectivity sparsity. Panels **(B–D)** show the scatter plots of node degrees between the two networks at sparsity level of 10% **(B)**, 20% **(C)**, and 30% **(D)**, respectively. The lines in the scatter plots represent the linear fit.

At all sparsity levels between 6 and 40%, the correlations between node degrees of the coactivation network and the resting-state network were small (range from 0.17 to 0.38) (Figure 4A). We then plotted the node degrees of the coactivation network against the node degrees of the resting-state network at 10, 20, and 30% sparsity levels (Figures 4B–D). We observed that there were several nodes in the upper right corner or lower right corner of the scatter plots, which indicates that these nodes had higher degrees in one network but not in the other network.

**Figure 5:**
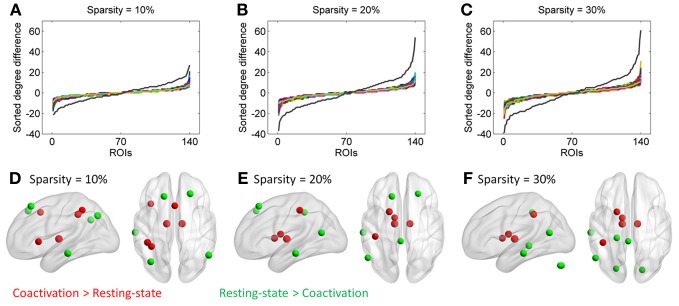
**Top row, sorted degree differences of 140 ROIs between the coactivation network and the resting-state network at sparsity of 10% (A), 20% (B), and 30%(C), respectively.** The chromatic lines represent sorted degree differences of scrambled networks for 1000 permutations. Bottom row, five regions that have largest and least degree differences between the coactivation network and the resting-state network at sparsity of 10% **(D)**, 20% **(E)**, and 30% **(F)**, respectively.

**Figure 6:**
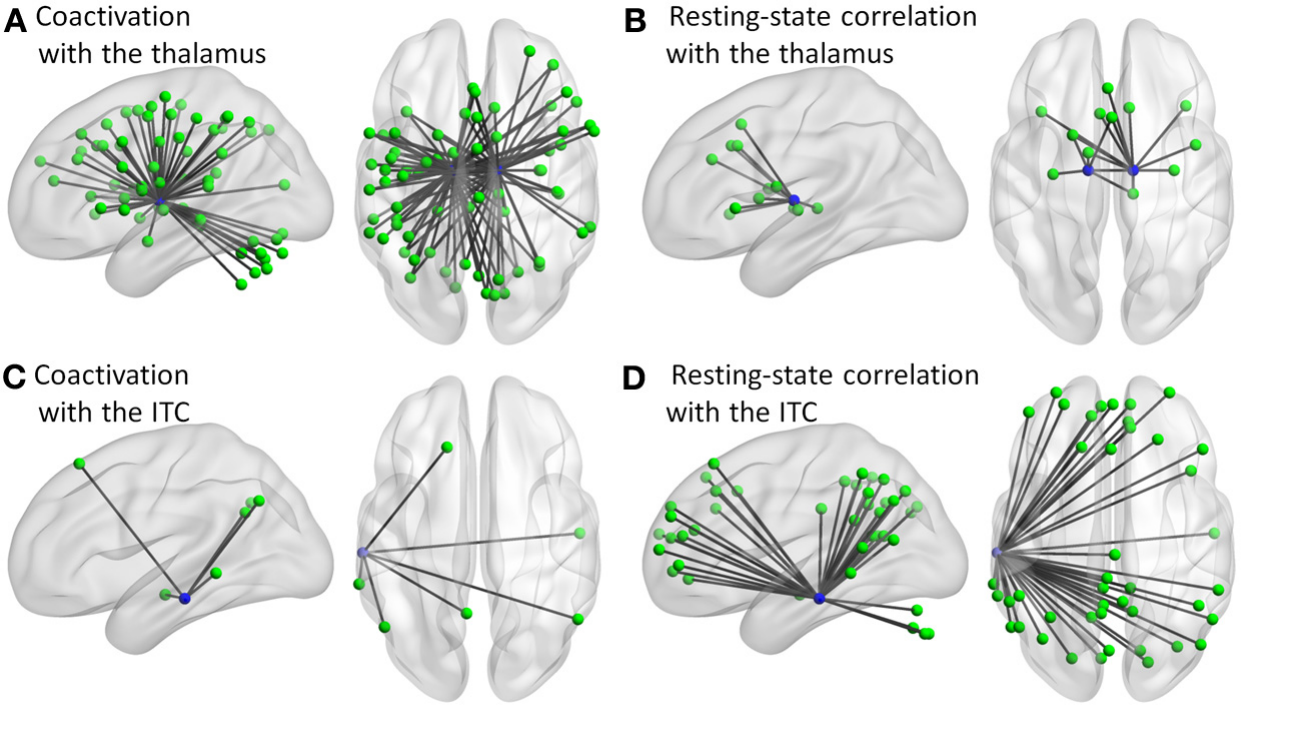
**Connectivity of the thalamus (A,B) and left inferior temporal cortex (ITC) (C,D) for the coactivation (A,C) and resting-state correlation (B,D) networks at sparsity of 20%**.

Additional analysis showed that the distribution of degree differences between the coactivation network and the resting-state network were outside the distribution of sorted degree differences of randomized 1000 permutations (Figures 5A–C), indicating that the degree differences between the two networks are not likely due to random noises. We then subtracted the degrees in the activation network by the degrees in the resting-state network for all 140 nodes at sparsity levels of 10, 20, and 30%, respectively. The top five nodes that had the greatest degree differences between the two networks are illustrated in Figures 5D–F and Table 1. Across the three sparsity levels, the bilateral thalamus demonstrated higher degrees in the coactivation network compared with the resting-state network. Other regions, including the basal ganglia, inferior parietal lobule (IPL), posterior parietal cortex, medial frontal cortex (mFC), and anterior insula, also showed higher degrees in the coactivation network at various sparsity levels. In contrast, a node in the inferior temporal cortex revealed consistently higher degree in the resting-state network compared with the coactivation network. Other regions, including the precuneus, angular gyrus, inferior parietal sulcus (IPS), temporoparietal junction (TPJ), superior frontal cortex, parahippocampal gyrus, and inferior cerebellum, also showed higher degrees in the resting-state network at various sparsity levels. The connectivity of the thalamus and the left inferior cortex for the two networks at sparsity level of 20% are illustrated in Figure 6.

**Table 1:** **Top five regions that have greater or smaller degree in the coactivation network as compared with in the resting-state network for the sparsity of 10, 20, and 30%, respectively**.

**MNI coordinates**			**Label**	**Degree differences Coactivation > correlation**
***x***	***y***	***z***		
**SPARSITY = 10%**				
**−12**	**−12**	**6**	**Thalamus**	**27**
**11**	**−12**	**6**	**Thalamus**	**24**
**−**41	**−**40	42	IPL	21
**−**35	**−**46	48	Post-parietal	18
0	15	45	mFC	16
**−**36	18	2	Ant insula	16
**−**16	29	54	Sup frontal	**−**18
**−**36	**−**69	40	IPS	**−**18
23	33	47	Sup frontal	**−**19
**−59**	**−25**	**−15**	**Inf temporal**	**−21**
51	**−**59	34	Angular gyrus	**−**21
**SPARSITY = 20%**				
**−12**	**−12**	**6**	**Thalamus**	**54**
**11**	**−12**	**6**	**Thalamus**	**42**
**−**20	6	7	Basal ganglia	36
**−**12	**−**3	13	Thalamus	30
**−**41	**−**31	48	Post-parietal	30
23	33	47	Sup frontal	**−**23
**−**3	**−**38	45	Precuneus	**−**24
**−**52	**−**63	15	TPJ	**−**26
**−**16	29	54	Sup frontal	**−**28
**−59**	**−25**	**−15**	**Inf temporal**	**−37**
**SPARSITY = 30%**				
**−12**	**−12**	**6**	**Thalamus**	**61**
**11**	**−12**	**6**	**Thalamus**	**52**
**−**12	**−**3	13	Thalamus	37
**−**41	**−**40	42	IPL	37
**−**20	6	7	Basal ganglia	34
18	**−**81	**−**33	Inf cerebellum	**−**26
**−**3	**−**38	45	Precuneus	**−**26
**−**21	**−**79	**−**33	Inf cerebellum	**−**26
**−**52	**−**63	15	TPJ	**−**31
**−**20	**−**33	**−**4	Parahippocampal	**−**31
**−59**	**−25**	**−15**	**Inf temporal**	**−39**

Regions highlighted in bold represent the regions show consistent differences between the two networks across the three sparsity levels.

## Discussion

The current study compared the whole brain network configurations between the coactivation network and the resting-state network. We first observed a high correlation between the coactivation strength and the resting-state correlation across all pairs of ROIs. In other words, if a pair of brain regions has greater functional connectivity in the resting-state, they are more likely to have greater coactivation, and vice versa. This is in line with previous findings that the coactivation patterns correspond well with the resting-state connectivity and networks (Toro et al., 2008; Smith et al., 2009). However, further analysis revealed substantial differences in network configuration between the two networks. Specifically, the coactivation network revealed higher global efficiency, lower mean clustering coefficient, and lower modularity as compared with the resting-state network. Shifts in hub regions were also observed where the thalamus had greater degrees in the coactivation network than in the resting-state network, and a region in the left inferior temporal cortex had greater degrees in the resting-state network than in the coactivation network. These results were similar when using NKI-dataset (see supplementary materials).

The brain network exhibits a so-called “small-world” property (Watts and Strogatz, 1998) that the network has greater mean local efficiency but smaller global efficiency than random network. Small world properties have been initially shown in non-human primates (Sporns, 2000; Stephan et al., 2000) and later in human brain network using both the resting-state fMRI (Salvador et al., 2005; van den Heuvel et al., 2009) and diffusion weighted imaging (Hagmann et al., 2007; Gong et al., 2009). The current results revealed greater global efficiency and smaller mean local efficiency for the coactivation network as compared with the resting-state network, suggesting that the whole brain is connected more efficiently to support global information flow during task performing. These results are in line with the findings that the brain exhibits higher global efficiency as task difficulty increases (Kitzbichler et al., 2011), and in the awake state compared with the stage 1 sleep (Uehara et al., 2013).

The current study also revealed smaller modularity in the coactivation network as compared with the resting-state network. These results suggest that the whole brain is less segregated as independent modules when performing tasks as compared with the resting-state. In other words, there are more between module connections and less within module connections when performing tasks, while more within module connections and less between module connections exist in the resting-state. These results are in line with the economy theory of brain network that long range between system connections are more costly, so that dynamic connectivity between brain systems is only present upon task demands (Bullmore and Sporns, 2012). Consistent with this notion, brain network modularity reduces when the task demand increases (Kitzbichler et al., 2011), and in awake state than during non-rapid eye movement sleep (Boly et al., 2012).

In addition to the whole brain network properties, the current study also identified hub regions by calculating degrees (number of connections) for each ROI. In contrast to the high correlation of network strengths between the coactivation network and the resting-state network, the correlations of node degrees between the two networks are small (around 0.3). This suggests a hub shift between task performance and resting-state (Fransson et al., 2011; Achard et al., 2012), which may reflect the adaptive brain reorganization that support the execution of tasks. However, the low correlations of degrees are inconsistent with a previous study showing a high correlation of degrees between a passive fixation condition and a continuous semantic classification task condition (Buckner et al., 2009). The differences may be due to the methodological differences used by Buckner et al. (voxel-wise analysis); the voxel-wise degree distributions are likely to be affected by the underlying brain anatomy, and the high correlation between the two degree maps may partially reflect the anatomical information. In addition, the differences may also be explained by the task adopted by Buckner et al., which is different from the current coactivation approach. Further studies are needed to investigate shifts in hubs elicited by different tasks.

The thalamus regions showed consistently higher degrees (the number of connections with other regions) in the coactivation network as compared with the resting-state network. The thalamus relays visual and auditory information gathered from the eyes and ears to the cerebral cortex (Hotta and Kameda, 1963). Different parts of the thalamus have intensive connections to wide spread cortical regions (Zhang et al., 2008; Eckert et al., 2012). In addition, the thalamus as a relay is important for corticocortical communication, and thus is suggested to be a potential hub for the brain function (Guillery, 1995). Previous resting-state fMRI studies occasionally identified the thalamus as a hub region (van den Heuvel et al., 2008), however, most of studies did not support this view (Achard et al., 2006; Buckner et al., 2009; Yan and He, 2011; Zuo et al., 2012). This may be because the centrality (as measured by degree or eigenvector centrality) of the thalamus is context specific (Lohmann et al., 2010; Gili et al., 2013). This is in line with the present result, and suggests that the thalamus mediates corticocortical communication during task, but this mediation is weakened in the resting-state.

In contrast, the left inferior temporal cortex region revealed higher degree in the resting-state network as compared with the coactivation network. This region is part of the DMN (Raichle et al., 2001; Buckner et al., 2008), which is generally deactivated during tasks (Shulman et al., 1997). Regions that are connected with the left inferior temporal cortex mostly constitute the DMN (Figure 6D). Consistent with previous studies of brain centrality (Achard et al., 2006; Buckner et al., 2009), the left inferior temporal cortex showed high centrality in resting-state. But, the current analysis also revealed that the degree is significantly less in the coactivation network. This may reflect the less involvement of this region during tasks as compared with the resting-state (Shulman et al., 1997).

By comparing network configurations of the coactivation network with the resting-state network, the current analysis provides insight on the different brain modes during task and resting-state. The brain during task exhibits greater small-worldness that facilitates global information transmission, and smaller modularity that facilitate information transmission between different systems. These results motivate future studies to investigate brain network configurations in different task conditions. In addition, the current analysis identified the thalamus as a hub region only in the coactivation network but not the resting-state network, suggesting that the role of thalamus in the brain network may be overlooked when studying the resting-state brain network. A difficulty of studying thalamus connectivity is that the thalamus is spatially heterogeneous, so that different substructures connect to different brain regions (Zhang et al., 2008; Eckert et al., 2012). Future studies may need to use fine spatial scales to investigate the thalamus and its effect on network configurations (Wang et al., 2009; Hayasaka and Laurienti, 2010).

Recently, several efforts have been made to study brain networks using inter-individual covariance from different imaging modalities, for example brain structures (Mechelli et al., 2005; Chen et al., 2008), brain metabolisms (Horwitz et al., 1984; Di et al., 2012), and resting-state brain parameters (Zhang et al., 2011; Taylor et al., 2012). Although these studies provide information on brain integration, the lack of theoretical basis causes difficulty in combining results from different imaging modalities. The current study may provide a theoretical framework to relate the different levels of brain network (e.g., anatomical, metabolic, and hemodynamic) in terms of local/global efficiencies and modular integrations. Future work on systematically comparing different levels of brain network configuration will facilitate in testing theories of brain organization such as the economic theory (Bullmore and Sporns, 2012).

### Conflict of interest statement

The authors declare that the research was conducted in the absence of any commercial or financial relationships that could be construed as a potential conflict of interest.
