# Supplementary material for: Task vs. rest—different network configurations between the coactivation and the resting-state brain networks
Source: Front Hum Neurosci. 2013 Sep 17;7:493. doi: 10.3389/fnhum.2013.00493 (PMC3775427; doi:10.3389/fnhum.2013.00493)
Supplement: Supplementary file 1 [file DataSheet1.DOCX]

**Supplementary Materials: Task vs. Rest - Different Network Configurations between the Coactivation and the Resting-State Brain Networks**

Xin Di, Suril Gohel, Eun H. Kim, Bharat B. Biswal

**Summary**

To examine whether the observed network property differences between the coactivation network and resting-state network were due to biased selection of the resting-state sample, we conduct the same analysis using another resting-state dataset to derive resting-state network, i.e. the Nathan Kline Institute (NKI) sample. The data analysis procedures were identical to the method implemented on Oulu dataset. We describe the characteristics of the NKI sample and report the results in the current supplementary materials. We also provide the full list of the regions of interest (ROIs) used in the present study (Table S1).

**The NKI sample**

The Nathan Kline Institute (NKI) / Rockland Sample from the INDI (international neuroimaging data-sharing initiative) Prospective Data Sharing Samples was used (<http://fcon_1000.projects.nitrc.org/indi/pro/nki.html>). This dataset originally contains 207 subjects, with a broad age range from 4 years to 85 years. We only included the subjects with age between 18 to 60 years. After discarding subjects with missing files and large head motion (greater than 3 mm), 106 subjects were finally included in the analysis (38 females). The mean age of the effective sample is 35.2 years (range from 18 to 57 years). 260 resting-state functional images were acquired for each subject (TR = 2.5 s, spatial resolution 3 x 3 x 3.33 mm^3^). High resolution anatomical image was also acquired for each subject using MPRAGE sequence (Magnetization Prepared Rapid Acquisition Gradient Echo). More information for the data can be found at the data webpage. The imaging processing steps were identical to those of Oulu dataset.

**Results for the NKI sample**

The pattern of the resting-state correlation matrix of NKI dataset (Figure S1B) was similar to the coactivation matrix (Figure S1A). The coactivation strengths and the resting-state correlation strengths among the 9,730 pairs of ROIs showed a strong correlation (*r = 0.77*) (Figure S1C). The coactivation network revealed greater global efficiency, smaller mean clustering coefficient, and smaller modularity compared with the resting-state network at selected sparsity levels (Figure S2). The direction of the differences in the network properties were the same as the Oulu dataset; but the significant differences occurred at different sparsity levels.

The correlations between node degrees of the coactivation network and the resting-state network were moderate (range from 0.36 to 0.45) (Figure S3A). The correlations were higher than those calculated using the Oulu dataset as a control network. The five nodes that had the greatest degree differences between the two networks are illustrated in Figure S3B-D and Table S2. Across the three sparsity levels, the bilateral thalamus and left inferior parietal lobule (IPL) demonstrated higher degrees in the coactivation network compared with the resting-state network. Other regions, including the medial frontal cortex (mFC), posterior parietal cortex, and basal ganglia, also showed higher degrees in the coactivation network at various sparsity levels. In contrast, the nodes that showed higher degree in the resting-state network were inconsistent across the three sparsity levels.

**Figure S1** Coactivation matrix (A), resting-state correlation matrix calculated from the NKI dataset (B), and the relationship between coactivation strengths and resting-state correlations (C). Each dot in the scatter plot represents one pair of ROIs. The red line in panel C represents the linear fit.


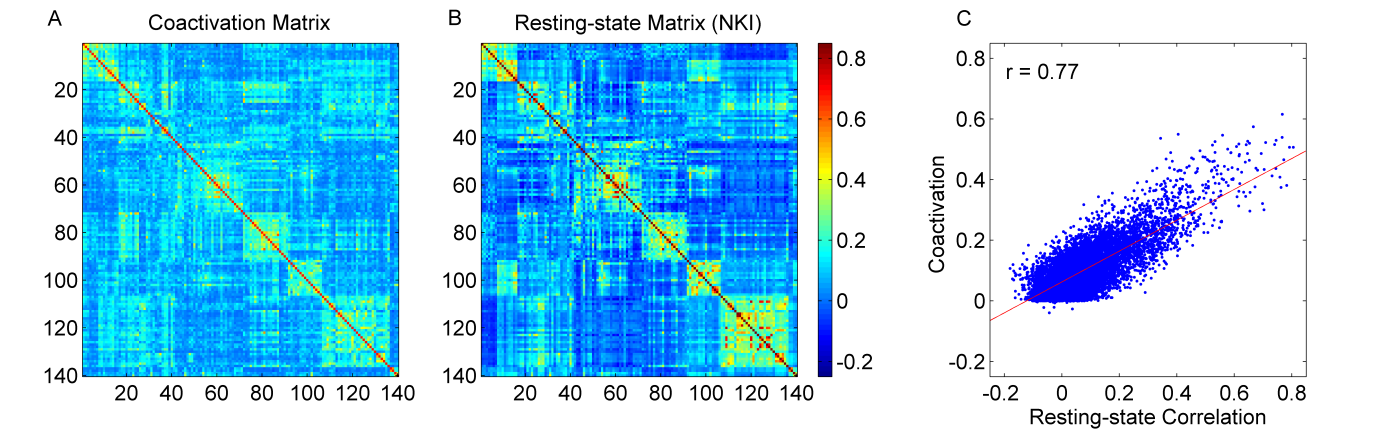


**Figure S2** Global efficiency (A), mean clustering coefficient (B) and modularity (C) for the coactivation, resting-state of the NKI dataset, and random networks as a function of connectivity sparsity. The shading areas represent significant differences between the coactivation and resting-state networks at p < 0.001 based on 1000 permutations.


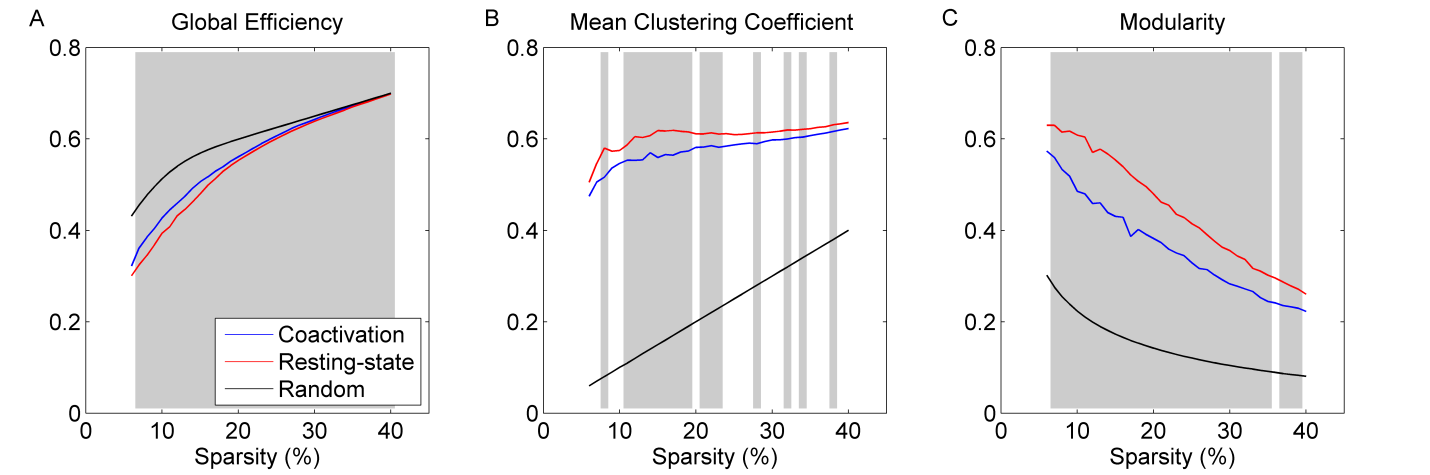


**Figure S3** A) Correlations between nodes' degree of the coactivation network and the resting-state network of the NKI dataset as a function of connectivity sparsity. Five regions that have the largest and least degree differences between the coactivation network and the resting-state network at sparsity of (B)10%, (C) 20%, and (D) 30%.


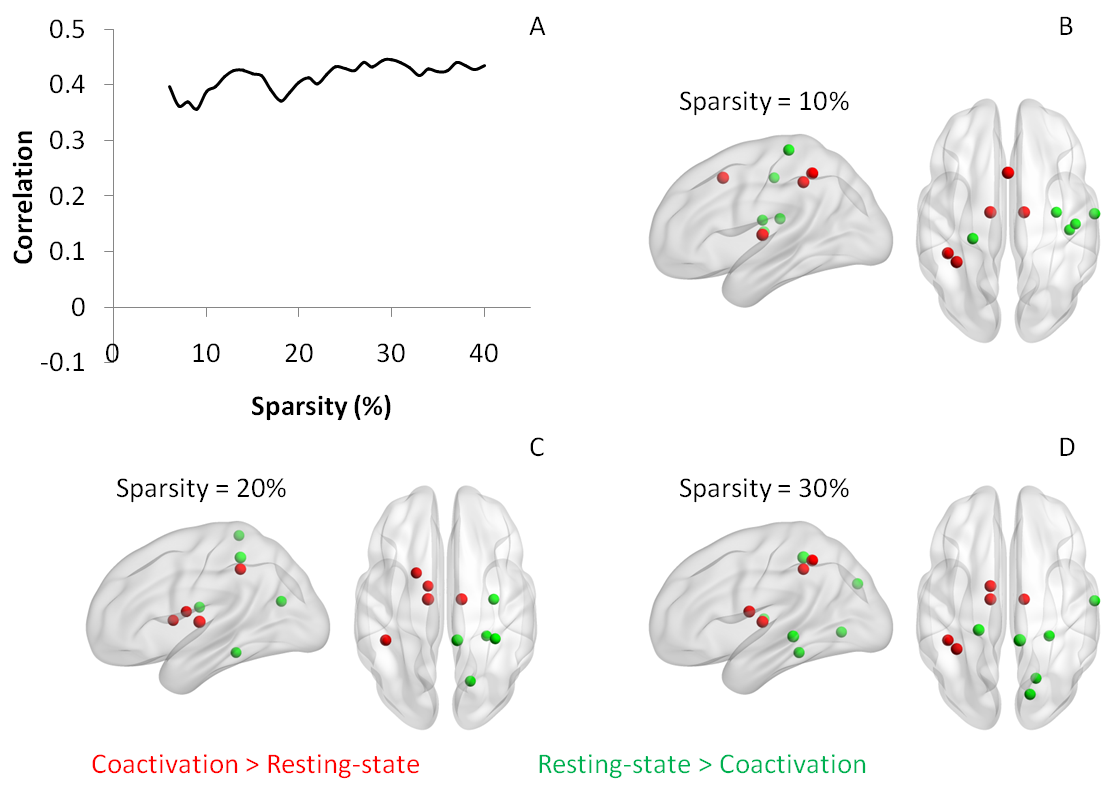


**Table S1** 140 functionally defined ROIs used in the current study.

| No | MNI coordinates | | | Label | Network |
| --- | --- | --- | --- | --- | --- |
|  | x | y | z |  |  |
| 1 | -34 | -67 | -29 | inf cerebellum | Cerebellum |
| 2 | 32 | -61 | -31 | inf cerebellum | Cerebellum |
| 3 | -37 | -54 | -37 | inf cerebellum | cerebellum |
| 4 | 18 | -81 | -33 | inf cerebellum | cerebellum |
| 5 | -6 | -79 | -33 | inf cerebellum | cerebellum |
| 6 | -21 | -79 | -33 | inf cerebellum | cerebellum |
| 7 | 33 | -73 | -30 | inf cerebellum | cerebellum |
| 8 | -24 | -54 | -21 | lat cerebellum | cerebellum |
| 9 | 21 | -64 | -22 | lat cerebellum | cerebellum |
| 10 | -28 | -44 | -25 | lat cerebellum | cerebellum |
| 11 | 14 | -75 | -21 | med cerebellum | cerebellum |
| 12 | 1 | -66 | -24 | med cerebellum | cerebellum |
| 13 | -6 | -60 | -15 | med cerebellum | cerebellum |
| 14 | -16 | -64 | -21 | med cerebellum | cerebellum |
| 15 | 5 | -75 | -11 | med cerebellum | cerebellum |
| 16 | -11 | -72 | -14 | med cerebellum | cerebellum |
| 17 | -2 | 30 | 27 | ACC | cingulo-opercular |
| 18 | 38 | 21 | -1 | ant insula | cingulo-opercular |
| 19 | -36 | 18 | 2 | ant insula | cingulo-opercular |
| 20 | 27 | 49 | 26 | aPFC | cingulo-opercular |
| 21 | -20 | 6 | 7 | basal ganglia | cingulo-opercular |
| 22 | -6 | 17 | 34 | basal ganglia | cingulo-opercular |
| 23 | 11 | -24 | 2 | basal ganglia | cingulo-opercular |
| 24 | 9 | 20 | 34 | dACC | cingulo-opercular |
| 25 | 0 | 15 | 45 | mFC | cingulo-opercular |
| 26 | 37 | -2 | -3 | mid insula | cingulo-opercular |
| 27 | -30 | -14 | 1 | mid insula | cingulo-opercular |
| 28 | 32 | -12 | 2 | mid insula | cingulo-opercular |
| 29 | -55 | -44 | 30 | parietal | cingulo-opercular |
| 30 | 58 | -41 | 20 | parietal | cingulo-opercular |
| 31 | -4 | -31 | -4 | post cingulate | cingulo-opercular |
| 32 | 8 | -40 | 50 | precuneus | cingulo-opercular |
| 33 | 42 | -46 | 21 | sup temporal | cingulo-opercular |
| 34 | 43 | -43 | 8 | temporal | cingulo-opercular |
| 35 | -59 | -47 | 11 | temporal | cingulo-opercular |
| 36 | -12 | -3 | 13 | thalamus | cingulo-opercular |
| 37 | -12 | -12 | 6 | thalamus | cingulo-opercular |
| 38 | 11 | -12 | 6 | thalamus | cingulo-opercular |
| 39 | -52 | -63 | 15 | TPJ | cingulo-opercular |
| 40 | -48 | 6 | 1 | vFC | cingulo-opercular |
| 41 | 51 | 23 | 8 | vFC | cingulo-opercular |
| 42 | 9 | 39 | 20 | ACC | default |
| 43 | -48 | -63 | 35 | angular gyrus | default |
| 44 | 51 | -59 | 34 | angular gyrus | default |
| 45 | -25 | 51 | 27 | aPFC | default |
| 46 | 28 | -37 | -15 | fusiform | default |
| 47 | -59 | -25 | -15 | inf temporal | default |
| 48 | -61 | -41 | -2 | inf temporal | default |
| 49 | 52 | -15 | -13 | inf temporal | default |
| 50 | -36 | -69 | 40 | IPS | default |
| 51 | 0 | 51 | 32 | mPFC | default |
| 52 | 45 | -72 | 29 | occipital | default |
| 53 | -9 | -72 | 41 | occipital | default |
| 54 | -28 | -42 | -11 | occipital | default |
| 55 | -2 | -75 | 32 | occipital | default |
| 56 | 10 | -55 | 17 | post cingulate | default |
| 57 | -11 | -58 | 17 | post cingulate | default |
| 58 | 1 | -26 | 31 | post cingulate | default |
| 59 | -5 | -52 | 17 | post cingulate | default |
| 60 | -5 | -43 | 25 | post cingulate | default |
| 61 | 5 | -50 | 33 | precuneus | default |
| 62 | 11 | -68 | 42 | precuneus | default |
| 63 | 9 | -43 | 25 | precuneus | default |
| 64 | -3 | -38 | 45 | precuneus | default |
| 65 | -6 | -56 | 29 | precuneus | default |
| 66 | 23 | 33 | 47 | sup frontal | default |
| 67 | -16 | 29 | 54 | sup frontal | default |
| 68 | -6 | 50 | -1 | vmPFC | default |
| 69 | 9 | 51 | 16 | vmPFC | default |
| 70 | -11 | 45 | 17 | vmPFC | default |
| 71 | 8 | 42 | -5 | vmPFC | default |
| 72 | -1 | 28 | 40 | ACC | fronto-parietal |
| 73 | 29 | 57 | 18 | aPFC | fronto-parietal |
| 74 | -29 | 57 | 10 | aPFC | fronto-parietal |
| 75 | -42 | 7 | 36 | dFC | fronto-parietal |
| 76 | 40 | 17 | 40 | dFC | fronto-parietal |
| 77 | 44 | 8 | 34 | dFC | fronto-parietal |
| 78 | 46 | 28 | 31 | dlPFC | fronto-parietal |
| 79 | -44 | 27 | 33 | dlPFC | fronto-parietal |
| 80 | -48 | -47 | 49 | IPL | fronto-parietal |
| 81 | -41 | -40 | 42 | IPL | fronto-parietal |
| 82 | -53 | -50 | 39 | IPL | fronto-parietal |
| 83 | 44 | -52 | 47 | IPL | fronto-parietal |
| 84 | 54 | -44 | 43 | IPL | fronto-parietal |
| 85 | -32 | -58 | 46 | IPS | fronto-parietal |
| 86 | 32 | -59 | 41 | IPS | fronto-parietal |
| 87 | -35 | -46 | 48 | post parietal | fronto-parietal |
| 88 | 42 | 48 | -3 | vent aPFC | fronto-parietal |
| 89 | -43 | 47 | 2 | vent aPFC | fronto-parietal |
| 90 | 39 | 42 | 16 | vlPFC | fronto-parietal |
| 91 | -52 | 28 | 17 | vPFC | fronto-parietal |
| 92 | -44 | -63 | -7 | occipital | occipital |
| 93 | 17 | -68 | 20 | occipital | occipital |
| 94 | 36 | -60 | -8 | occipital | occipital |
| 95 | -34 | -60 | -5 | occipital | occipital |
| 96 | 39 | -71 | 13 | occipital | occipital |
| 97 | 19 | -66 | -1 | occipital | occipital |
| 98 | 9 | -76 | 14 | occipital | occipital |
| 99 | 15 | -77 | 32 | occipital | occipital |
| 100 | -29 | -75 | 28 | occipital | occipital |
| 101 | 20 | -78 | -2 | occipital | occipital |
| 102 | -18 | -50 | 1 | occipital | occipital |
| 103 | 29 | -81 | 14 | post occipital | occipital |
| 104 | 33 | -81 | -2 | post occipital | occipital |
| 105 | -37 | -83 | -2 | post occipital | occipital |
| 106 | 46 | -62 | 5 | temporal | occipital |
| 107 | 60 | 8 | 34 | dFC | sensorimotor |
| 108 | 58 | 11 | 14 | frontal | sensorimotor |
| 109 | 53 | -3 | 32 | frontal | sensorimotor |
| 110 | -42 | -3 | 11 | mid insula | sensorimotor |
| 111 | -36 | -12 | 15 | mid insula | sensorimotor |
| 112 | 33 | -12 | 16 | mid insula | sensorimotor |
| 113 | -26 | -8 | 54 | parietal | sensorimotor |
| 114 | -47 | -18 | 50 | parietal | sensorimotor |
| 115 | -38 | -15 | 59 | parietal | sensorimotor |
| 116 | 46 | -20 | 45 | parietal | sensorimotor |
| 117 | -55 | -22 | 38 | parietal | sensorimotor |
| 118 | -24 | -30 | 64 | parietal | sensorimotor |
| 119 | 41 | -23 | 55 | parietal | sensorimotor |
| 120 | -47 | -12 | 36 | parietal | sensorimotor |
| 121 | 42 | -24 | 17 | post insula | sensorimotor |
| 122 | -41 | -31 | 48 | post parietal | sensorimotor |
| 123 | 10 | 5 | 51 | pre-SMA | sensorimotor |
| 124 | -54 | -9 | 23 | precentral gyrus | sensorimotor |
| 125 | 44 | -11 | 38 | precentral gyrus | sensorimotor |
| 126 | -44 | -6 | 49 | precentral gyrus | sensorimotor |
| 127 | 46 | -8 | 24 | precentral gyrus | sensorimotor |
| 128 | 58 | -3 | 17 | precentral gyrus | sensorimotor |
| 129 | 0 | -1 | 52 | SMA | sensorimotor |
| 130 | 34 | -39 | 65 | sup parietal | sensorimotor |
| 131 | -53 | -37 | 13 | temporal | sensorimotor |
| 132 | -41 | -37 | 16 | temporal | sensorimotor |
| 133 | 59 | -13 | 8 | temporal | sensorimotor |
| 134 | -54 | -22 | 9 | temporal | sensorimotor |
| 135 | 43 | 1 | 12 | vFC | sensorimotor |
| 136 | -55 | 7 | 23 | vFC | sensorimotor |
| 137 | 20 | -4 | -15 | Amygdala | additional |
| 138 | -20 | -6 | -15 | Amygdala | additional |
| 139 | -20 | -33 | -4 | Parahippocampal | additional |
| 140 | 14 | -33 | -7 | Parahippocampal | additional |

**Table S2** Top five regions that have greater or smaller degree in the coactivation network as compared with in the resting-state network of the NKI dataset for the sparsity of 10%, 20%, and 30%, respectively. Regions highlighted in bold represent the regions show consistent differences between the two networks across the three sparsity levels.

| MNI coordinates | | | Label | Degree differences  Coactivation > correlation |
| --- | --- | --- | --- | --- |
| x | y | z |  |  |
| Sparsity = 10% | | |  |  |
| **-12** | **-12** | **6** | **thalamus** | **23** |
| 0 | 15 | 45 | mFC | 22 |
| -35 | -46 | 48 | post parietal | 21 |
| **11** | **-12** | **6** | **thalamus** | **20** |
| **-41** | **-40** | **42** | **IPL** | **20** |
| 46 | -20 | 45 | parietal | -13 |
| 33 | -12 | 16 | mid insula | -14 |
| 42 | -24 | 17 | post insula | -15 |
| -24 | -30 | 64 | parietal | -16 |
| 59 | -13 | 8 | temporal | -16 |
| Sparsity = 20% | | |  |  |
| **-12** | **-12** | **6** | **thalamus** | **43** |
| **11** | **-12** | **6** | **thalamus** | **35** |
| **-41** | **-40** | **42** | **IPL** | **29** |
| -20 | 6 | 7 | basal ganglia | 26 |
| -12 | -3 | 13 | thalamus | 25 |
| 28 | -37 | -15 | fusiform | -22 |
| 17 | -68 | 20 | occipital | -23 |
| 34 | -39 | 65 | sup parietal | -23 |
| 33 | -12 | 16 | mid insula | -24 |
| 8 | -40 | 50 | precuneus | -32 |
| Sparsity = 30% | | |  |  |
| **-12** | **-12** | **6** | **thalamus** | **39** |
| **-12** | **-3** | **13** | **thalamus** | **37** |
| -35 | -46 | 48 | post parietal | 35 |
| **-41** | **-40** | **42** | **IPL** | **31** |
| 11 | -12 | 6 | thalamus | 30 |
| 19 | -66 | -1 | occipital | -26 |
| 59 | -13 | 8 | temporal | -26 |
| 28 | -37 | -15 | fusiform | -27 |
| 15 | -77 | 32 | occipital | -28 |
| -20 | -33 | -4 | Parahippocampal | -36 |
| 8 | -40 | 50 | precuneus | -45 |
